# The Vortex-Slurry Implementation: A Cheap, Easy, and Ultrafast Mechanochemical Tool to Synthesize/Screen Pharmaceutical Salts and Cocrystals

**DOI:** 10.1021/acsomega.5c02408

**Published:** 2025-09-10

**Authors:** Paulo N. de Souza, Lucas V. C. Militão, Pollyana P. Firmino, Pedro H. de O. Santiago, João H. de Araujo-Neto, Javier Ellena, Cecilia C. P. da Silva

**Affiliations:** † Department of Physics and Interdisciplinar Science, 117186 Universidade de São Paulo, Instituto de Física de São Carlos, Avenida Trabalhador Sãocarlense, 400, Centro, LaMuCrEs, São Carlos SP BR 13560-970, Brazil; ‡ Dipartimento di Chimica “Giacomo Ciamician”, Università Di Bologna, Via Selmi, 2, Bologna Emilia-Romagna 40126, Italy; § Instituto de Química, Departamento de Quimica Fundamental, Universidade de São Paulo Av. Prof. Dr. Lineu Prestes, 748, São Paulo BR 05508-900, Brazil

## Abstract

The vortex-slurry implementation is a combination of the best insights taken from multiple solid-state methods using accessible tools to improve the physical scenario, mainly designed for the supramolecular synthesis of new multicomponent pharmaceutical solid forms by coupling mechanochemistry and slurry techniques within a vortex mixer. The proposal of the vortex-slurry implementation is to provide a totally different experimental ambient capable of reaching new 3D thermodynamic states, not allowed in the current commercial mechanochemistry methods (1D mixer mill and 2D planetary mill). The obtained compounds could be easily scaled from milligrams to grams, thus opening the door for the possibility of an industrial scaling approach. The improvements achieved by this new implementation were validated by resynthesizing already reported salts and cocrystals and by presenting a new solid form for the antiviral drug Stavudine (DT4), which is an orally administered second-line drug in HIV treatment. Theoretical studies revealed that this implementation enhances the activation energy of the system due to the ball bearings’ helical movements. It is expected that this implementation can spread to the scientific community, allowing manufacturability of new drug candidates and generating improvements in the quality of life of patients.

## Graphical abstract

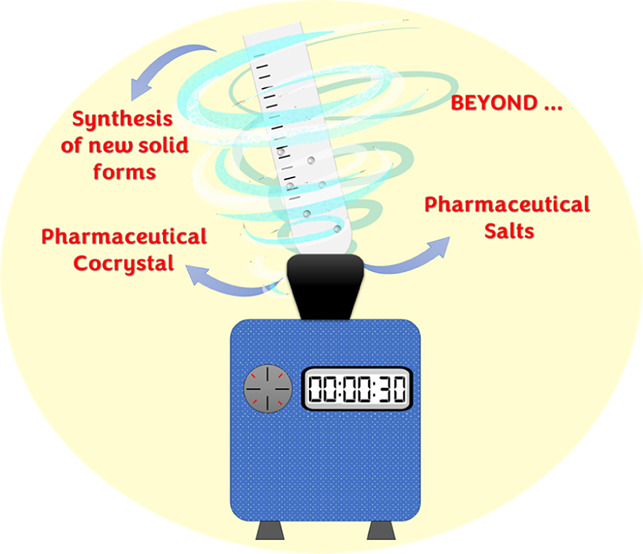

## Introduction

One of the main challenges in the University–Pharmaceutical Industry interface is the manufacturability of the new components obtained/synthesized in the academic laboratories, with reaction time, yield, and the number of steps involved normally not suitable for the industry requirements.^1–4^ At the laboratory, most of the new compounds, in particular salts and/or cocrystals of Active Pharmaceutical Ingredients (APIs), are obtained by slow evaporation from solvents.^5–8^ Although this is a very efficient technique, in terms of generating new products, the solvent amount needed to scale up such experiments becomes a major obstacle for implementing them in an industrial-scale production.^2,9,10^ Trying to address this problem, an alternative cocrystallization technique, named as slurry, has been developed and applied.^11,12^ It consists of adding a minimal amount of solvent in order to turn the system wet enough to be submitted to a constant shaking (usually at 80 rpm), under different temperatures, aiming to provide collision between the particles (and thus, nucleation and the formation of new compounds), until the complete evaporation of solvent. Although very useful, the slurry technique requires at least 24–48 h for complete solvent evaporation, which also hinders its implementation at industrial levels.^13–15^ In addition, although less solvent is required in comparison with the slow evaporation technique, for scaling-up the new compounds via slurry, solvent amount can still be an issue, beyond time as mentioned.^5^

To circumvent the problem, scientists have applied mechanochemical methods. However, even when mechanochemistry has been used since long time for obtaining products via corubbing two or more materials, just recently started to be applied into the pharmaceutical field.^5,16^ In this method, with or without the use of a small amount (drops) of solvent, it is possible to obtain new compounds via chemical reactions induced by mechanical energy. Commercially available, there are two main types of automatic mechanochemical apparatuses: the mixer mill and the planetary mill. Although both are used for grinding and mixing materials, by utilizing ball bearings to generate mechanical forces for the reagents, they differ significantly in their design, operation, and application. The mixer mill operates on a simpler mechanism where the grinding jars, usually small, move in a single axis (1D movement). Mixer mills have been more used at laboratories to perform cocrystallization experiments once they are easy to handle, require small amounts of the reactants (milligrams to a few grams), and offer the ability to control temperature during the grinding process. Nevertheless, their limitation lies in the scale-up and screening of the reaction conditions. On the other hand, planetary ball mills operate on a more complex mechanism where the grinding jars move in a dual axis (2D movement). Planetary mills have been more used by the industry to perform ultrafine grinding experiments because they are capable of providing high energy to the system when operating with larger jar volumes (grams to kilograms). However, their limitations are related to the need of external cooling systems, since they usually do not offer a built-in temperature control, as well as their fixed speed ratio, which constrains the ability to fine-tune the kinetic energy. In addition, it is worth mentioning that both of the above-mentioned systems allow the use of solvents, but by considering that they operate with totally sealed jars, the amount of solvent added may become an issue. Liquid-assisted grinding (LAG) experiments performed with automatic mechanochemical apparatus may have mechanochemistry results affected depending on the parameter η, i.e., the ratio of liquid added to the system in relation to the weight of the reactants. Empirically, for η = 0 μL/mg, neat grinding takes place, for η in the range of 0–2 μL/mg, LAG takes place, for 2 μL/mg < η < 12 μL/mg, slurry takes place, and for η > 12 μL/mg, a typical solution reaction takes place. In this way, if η lies in the range of 2 μL/mg, it may result in the formation of a slurry, that requires time to evaporate and may not provide the desired cocrystallization at the end. Thus, although many new pharmaceutical salts and cocrystals have been obtained via mechanochemical grinding, at the best of our knowledge, it is not an easy task, once finding the greatest time/frequency pair that will provide the crystalline new materials is not trivial.^17–27^

In this sense, aiming to contribute to the subject, our group developed an implementation by combining the best insights taken from multiple solid-state methods using accessible tools to improve the physical scenario for mainly obtaining new pharmaceutical cocrystals/salts by coupling mechanochemistry and slurry techniques within a vortex mixer. The proposal of the vortex-slurry implementation was to provide a totally different experimental ambient capable of reaching a new 3D thermodynamic state, not allowed in the commercial mechanochemistry apparatus mentioned above, and to evaluate its advantages over the already used ones. Herein, the setup is closer to planetary mills, as it provides an energy landscape better described by the complex movement combinations introduced by such a method. But, it also provides another degree of freedom, as the microcrystals also reach a vertical travel. Therefore, this implementation was planned as a way to improve collisions and energy delivery and accelerate the mechanochemical method. A detailed description of our implementation is performed in the Materials and Methods section. Also, to validate this implementation, we have selected from the literature a drug–coformer cocrystal, a drug–drug cocrystal, and a drug salt obtained by mechanochemistry and/or slurry, to provide a valid comparison of the improvements of the vortex-slurry implementation concerning reaction time and/or scale-up as well as with and without the use of solvents/ball bearings. Finally, to be able of exploring the potential of the method and have further information to perform a theoretical analysis of the system, we have obtained a new hydrate cocrystal of the antiviral drug Stavudine (DT4) with L-proline, a coformer belonging to the Generally Regarded as Safe (GRAS) list.^28^

## Experimental Section

### The “Vortex-Slurry Method” Applied to Crystal Engineering

First of all, it is necessary to introduce the method and provide insights into why it promotes cocrystallization. The three main necessary materials to perform the experiment are simple and depicted in Figure 1. They are: (1) a common vortex mixer, which undergoes until at least 3000 rpm (Figure 1a), (2) tapered-shaped vials, such as Falcon tubes or Eppendorfs, but not restricted to them (Figure 1b), and (3) ball bearings of different sizes and materials (Figure 1c).

**Figure 1:**
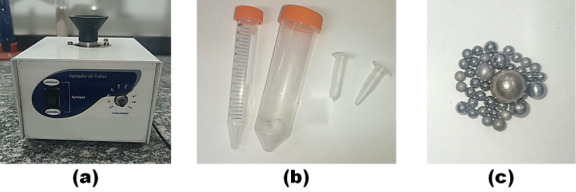
Materials necessary for the “Vortex-slurry Method” experiment, to determine (a) a common vortex mixer, (b) tapered-shaped vials, and (c) ball bearings of different sizes and materials.

Taking into account the principles of the available ball milling equipment (mixer and planetary), in the *vortex-slurry method* there are only two differences, but those have a huge impact on time and yield, concerning the synthesis of pharmaceutical salts and/or cocrystals. One of them is the fact that the Falcon tube (an Eppendorf can be used as well) is not completely sealed, since for experiments a 3D cap was printed containing holes large enough for the solvent to leave the system, but small enough for the ball bearings not to be projected out. This “opened” option is not allowed for the mixer ball milling, nor the planetary one, of course for security issues, but it can be done in the vortex-slurry method, since the experiment is performed in a chemical hood (Figure 1). The second difference is related to the geometry of the tubes utilized, which have a tapered shape, thus promoting a different pattern of movements for the ball bearings: a *hurricane* movement, or a *vortex* one. Basically, this movement is a combination of both movements observed in the mixer (translational) and planetary (rotational) ball milling equipment, being mainly generated due to the tapered shape of the flask. During their motion inside the tube, these balls hit each other and decay back to the bottom of the tube (the tapered region), beginning the movement again. In other words, the ball bearings end up mimicking the vortex creation that is obtained if a liquid, for example, is placed inside the vial, with the difference that they go up and down the tube as a result of translational shocking. To promote a maximum movement of these balls in the system, it is possible to move the tapered part of the tube so as to touch the walls of the cup head of the vortex mixer. For not tapered-shaped vials, a single circular movement is observed, such as the one for planetary ball milling.

As a result of this vortex motion of the ball bearings, up and down the tube (3D movement), when a bigger amount of solvent is added, to perform the slurry experiment, for example, the solvent is forced to evaporate quicker. As the system can operate open inside a chemical hood, then evaporation is favored, and the result occurs in a few minutes, instead of hours. In the same way, if no solvent or a few drops are added, the vortex motion of the ball bearings also allows a faster cocrystallization when compared with the available ball millings. An example of its use is depicted in Figure 2. The theoretical explanation concerning why cocrystallization is faster and the yield is higher in the vortex-slurry method is provided in a proper section.

**Figure 2:**
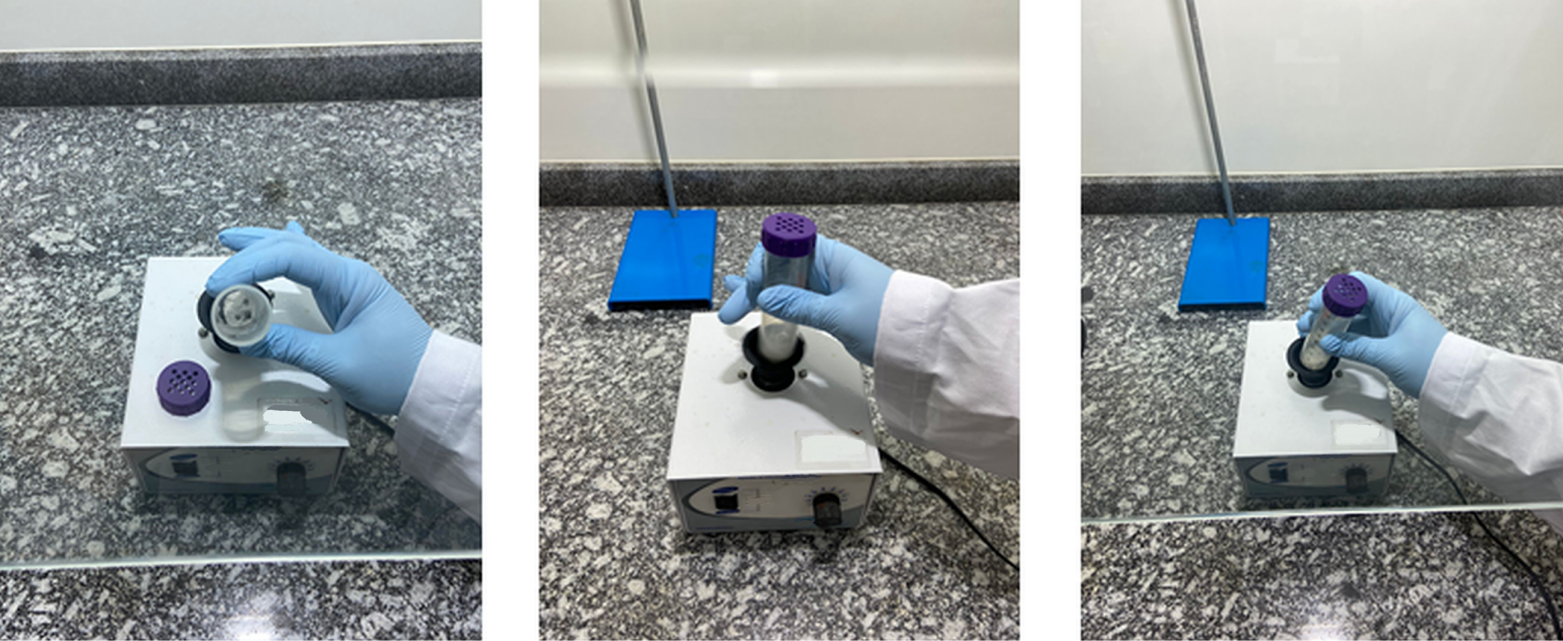
Example of how to easily use the “Vortex-slurry Method” with a Falcon tube and stainless ball bearings inside a chemical hood.

### Method Validation Experiments

Three experiments were selected to be reproduced herein: a cocrystal of hydrochlorothiazide and nicotinamide (API + GRAS),^29–31^ a drug–drug cocrystal of Meloxicam and Aspirin (API + API),^32^ and a salt of Ethionamide and maleic acid (API + GRAS).^22^

### Supramolecular Synthesis Performed by the “Vortex-Slurry Method”

For all the above-mentioned experiments, stoichiometric amounts of the reactants were weighed and placed into a Falcon tube or an Eppendorf vial. For the experiments performed with Hydrochlorothiazide (0.100 g) and nicotinamide (0.041 g), methanol (100 μL) was used as the solvent, as reported in the original work describing this API/coformer cocrystal.^29–31^ For the experiments performed with Meloxicam (0.905 g/0.182 g) and Aspirin (0.452 g/0.0966 g), tetrahydrofuran (3 mL) and chloroform (40 μL) were used as solvents, as reported in the original work describing this cocrystal, via slurry and mechanochemistry, respectively.^32^ For the experiments performed with Ethionamide (0.010 g, 0.06 mmol) and maleic acid (0.007 g), ethanol (20 and 100 μL) was used as the solvent, as reported in the original work describing this salt.^22^

### Synthesis of DT4 Hydrated Cocrystal with L-Proline (DT4·Lpro·H_2_O)

For the vortex-slurry experiment, powders of the raw materials and solvent (0.224 g of DT4, 0.115 g of L-pro, 15 μL ethanol) were first added into an Eppendorf containing two ball bearings. The system was shaken for 4 min (the time when the system looked dry), and the final powder was submitted to a powder X-ray diffraction experiment. In sequence, the powder was submitted to recrystallization in ethanol. The beaker was semicovered with Parafilm and left at 4 °C for 6 days, generating small single crystals suitable for single-crystal X-ray analysis. In addition, the experiment was reproduced in a Falcon tube, utilizing 2.240 g of DT4, 1.150 g of L-pro, and 1.5 mL of ethanol, with four ball bearings. The system was shaken during the third 6 min. This was the time when it was verified that the system looked dry.

### Single Crystal X-ray Diffraction Analysis of DT4·Lpro·H_2_O

The X-ray diffraction data collection for DT4·Lpro·H_2_O was performed at 100 K, using the MANACA beamline of the Brazilian National Synchrotron Light Laboratory (LNLS, Campinas-SP, Brazil), equipped with a Pilatus M2 detector and an MK3 mini-kappa, using phi-scans with 360° rotation in steps of 0.3° and an X-ray wavelength was regulated to 0.67019 Å. XDS software^33^ was employed for unit cell refinement, data collection and reduction, and empirical absorption correction. The structure was solved with the SHELXT program, using the Intrinsic Phasing method,^34^ while the non-hydrogen atoms were refined by the least-squares minimization method on F^2^ considering anisotropic displacement parameters and using the SHELXL program,^35^ both within the Olex2 program.^36^ Hydrogen atoms were positioned at calculated positions and refined with the riding model. Olex2 ^36^ was also used to prepare the graphical illustrations together with the Mercury software.^37,38^ Data collection and refinement parameters are listed in Table 2.

Crystallographic data of DT4·Lpro·H_2_O in CIF format have been deposited at the Cambridge Crystallographic Data Centre with deposition number 2306378. Copies of the data can be obtained free of charge from www.ccdc.cam.ac.uk.

### Powder X-ray Diffraction Experiments

PXRD analysis was performed for all of the samples obtained by the vortex-slurry cocrystallization method. The samples were packed in a cavity-type sample holder and pressed to avoid the preferred orientation. The X-ray powder diffraction data were all collected at room temperature on a Rigaku Ultima IV diffractometer equipped with a high-speed linear detector, D/TEX ULTRA, in Bragg–Brentano reflective geometry, with CuKα radiation and a Ni filter. The diffractograms were acquired in the 3–50° 2θ range with a step width of 0.02° and a constant counting time of 5° min^–1^. Full-profile phase analysis was performed using SmartLab Studio II 4.0 software (Rigaku Corporation, Japan) using the Rietveld method to check the phase purity of bulk samples^39^

Calculated diffractograms of the previously reported structures, found in Cambridge Structural Database (CSD)^40^ under refcodes *PIRXUL*,^29^ *ARIFOX*,^32^ and *WUVXUI* ^22^ were used from the CIF files deposited in the CSD,^40^ available free of charge and according to license agreement at www.ccdc.cam.ac.uk.

### Thermal Analysis

The thermogravimetric experiments for DT4·Lpro·H_2_O were performed using a Shimadzu TGA-50 thermobalance. 4.0 mg of sample was used for the measurement in an alumina pan and heated until 350 °C at 10 °C min^–1^ rate, under a N_2_ atmosphere (50 mL min^–1^). TGA experiments were not conducted for the other samples because their thermal behavior was previously reported. DSC data acquisition for DT4·Lpro·H_2_O and the other samples was carried out according to previous TGA data, that is, until the degradation temperature of the compound. These experiments were performed on a Shimadzu DSC-60 calorimeter. The samples were heated to 200 °C, with a rate of 10 °C min^–1^ in a crimped, sealed aluminum pan, using nitrogen as the purge gas (50 mL min^–1^). The data processing was done using Shimadzu TA-60 thermal data analysis software.

## Results and Discussion

### Validation of the Method

To validate the proposed vortex-slurry method, we have previously chosen representative cases from the literature reported by experienced scientists in the field of each example that was necessary to explore (drug–coformer cocrystal, drug–drug cocrystal, drug salt), capable of providing parameters to compare the improvements (or not) relative of applying the vortex-slurry methodology in reaction time and/or scale-up, as well as green chemistry.^29,42–45^ In this way, all the selected reported experiments have used solvent evaporation for crystal growth and some mechanochemical/slurry method for scale-up. The formation of these pharmaceutical salts and cocrystals was confirmed after the vortex-slurry experiments by using powder X-ray diffraction and Rietveld refinement. For this purpose, we selected the following compounds: an API-GRAS cocrystal of hydrochlorothiazide/nicotinamide,^29–31^ an API-API cocrystal of Meloxicam/Aspirin,^32^ and an API-GRAS salt of Ethionamide/maleic acid.^22^ For all of the experiments, four validation methodologies were applied: (1) no solvent/no ball bearings, (2) no solvent/ball bearings, (3) solvent/no ball bearings, and (4) solvent/ball bearings. These four combinations were conducted to investigate if the method could also generate the salts/cocrystals without needing to know solvent and ball bearings.

#### Hydrochlorothiazide/Nicotinamide (HCT·NIC) Cocrystal

Sanphui et al. were the first ones to report the HCT·NIC cocrystal,^29,30^ being the same obtained by grinding equimolar amounts of the raw materials (0.33 mmol) in a mortar and pestle for 15 min in the presence of a few drops of MeOH. Recently, the same cocrystal was obtained by Narala et al.^31^ via the hot-melt extrusion method. Therefore, the cocrystal was reproduced in the vortex method using the above four methods with time tracking to determine how quickly the cocrystal was formed via the proposed methodology. Equimolar amounts of the raw materials were used in all of the experiments. The solvent used was the same used by Sanphui et al.,^29,30^ MeOH. To compare, in the Narala et al.^31^ paper, the HCT·NIC (0.66 mmol) cocrystal was also reproduced via liquid-assisted grinding in a mortar and pestle, by utilizing 100 μL of MeOH and grinding by 20 min. Figure 3 shows the PXRD diffractograms obtained by the four experiments: none solvent/balls, solvent/no balls, balls/no solvent, and solvent/balls. The experimental diffraction patterns were compared with the calculated one generated from the reported crystalline structural data of HCT·NIC deposited in the CSD^40^ under the refcode PIRXUL.^29^ Each experiment in the vortex took a total of 3 min at a frequency of 3000 rpm. As can be seen, the HCT·NIC cocrystal was obtained in two of the four validation methods: solvent/no balls and solvent/balls. This is indicative that the method can also be employed in a simpler way. As to confirm the observed full conversion,Figure S1, additional thermal analysis of the synthesized cocrystal was performed (melting point 171.49 ± 2 °C), agreeing with the values found by Sanphui et al.^29^ (melting point 173 °C), and the Rietveld refinement phase purity (Figure 4).

**Figure 3:**
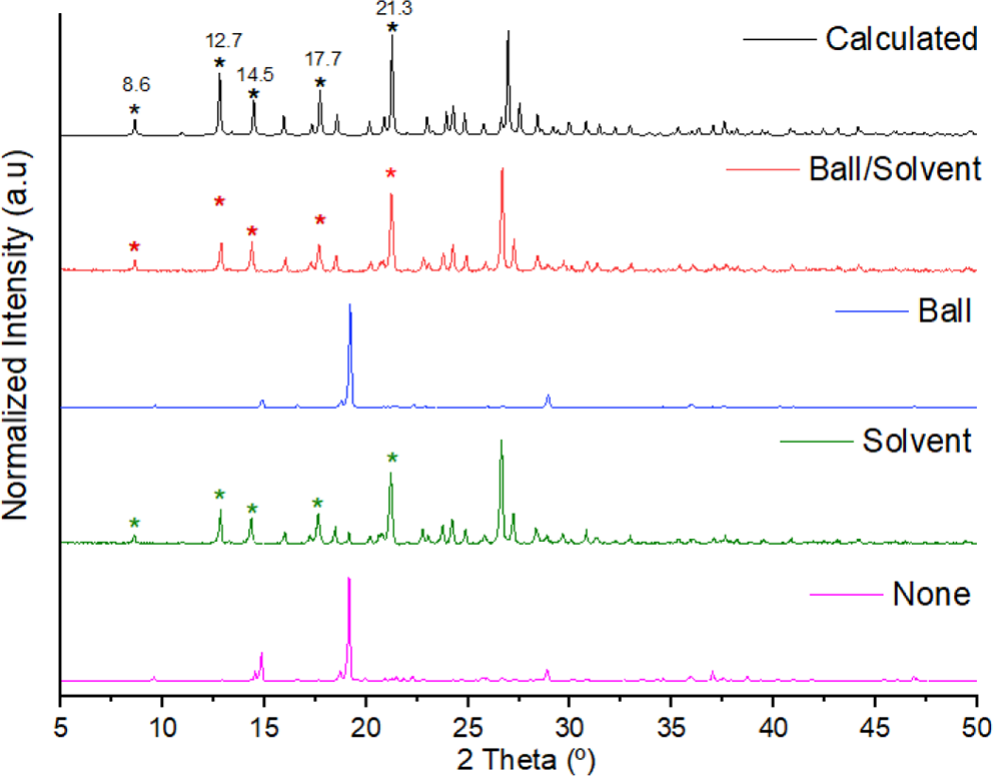
PRXD diffractograms for the validation of the vortex method by reproducing the HCT·NIC cocrystal (black). The validation experiments were performed using none balls/solvent (pink), no balls/solvent (green), balls/no solvent (blue), and balls/solvent (red).

**Figure 4:**
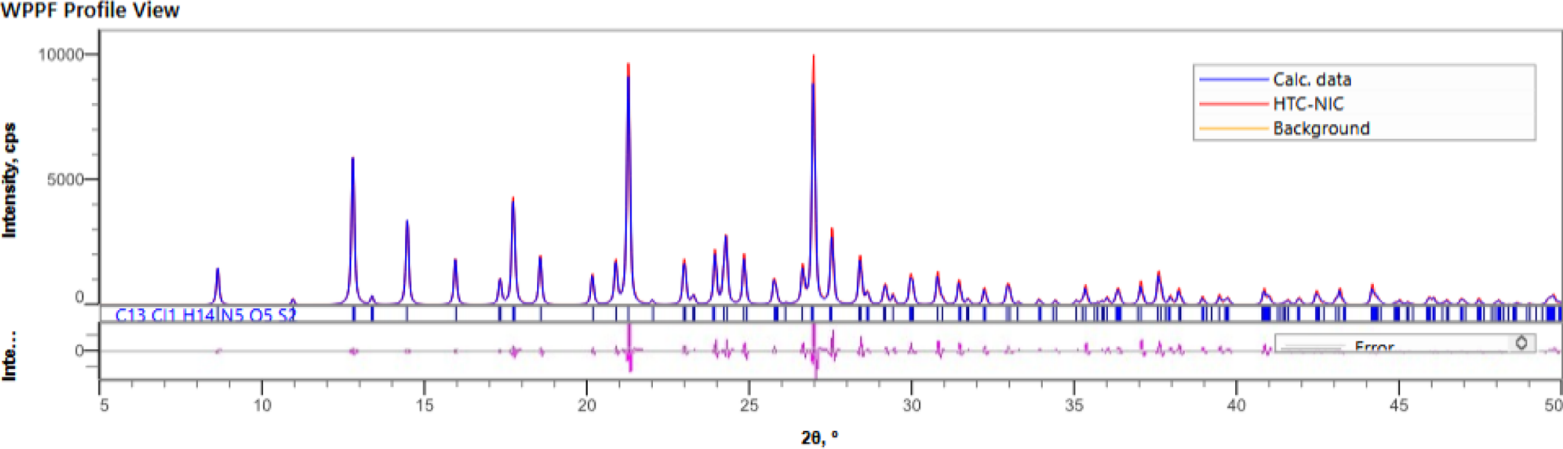
Rietveld refinement plots of the HCT·NIC cocrystal. The blue trace in the plots represents the experimental pattern, the green trace for calculated profile, the orange trace for background, the magenta trace represents the residual between the calculated and observed patterns, and the tick marks are indicative of *hkl* values of the crystal structures. The three samples are found to be phase pure.

#### Meloxicam/Aspirin (MEL·AAS) Cocrystal

Cheney et al. reported this cocrystal, successfully prepared by solution, slurry, and solvent-drop grinding methods.^32^ It was a very interesting work to compare with because it was possible to explore not only the four validation steps but also slurry and scale-up from milligrams to grams. For the validation method, Eppendorf tubes were used instead of the Falcon tubes. Equimolar amounts of MEL (0.1 mmol) and AAS (0.1 mmol) were used, and the chosen solvent for validation and scale-up was chloroform. For the slurry experiment using the vortex method, the same reported experiment was reproduced: 0.905 g (2.56 mmol) of MEL and 0.452 g (2.51 mmol) of AAS were slurred in 3 mL of tetrahydrofuran (THF), except by the fact that the Falcon tube was not sealed and 3000 rpm was applied in the vortex. Figure 5 exhibits the PXRD diffractograms for each experiment. Time was also counted to compare to the reported data. The experimental diffraction patterns were compared with the calculated ones generated from the reported crystalline structural data deposited in the CSD^40^ under the refcode ARIFOX.^32^ Each validation experiment in the vortex took a total of 5 min to be read using a frequency of 3000 rpm. As can be seen, the MEL·AAS cocrystal was also obtained in two of the four validation methods: no solvent/balls and solvent/balls. This is indicative that the method can work when only one of the two variables is applied, with 100% conversion when the solvent/balls system is used. Also, by utilizing balls and no solvents (blue diffractogram in Figure 5), peaks of the cocrystal can be seen (marked with an asterisk), together with peaks from the physical mixture of MEL and AAS, which are marked with black arrows in Figure 5. To confirm the full conversion additional thermal analysis of the synthesized cocrystal was performed (melting point 166.75 ± 2 °C, Figure S2) agreeing with the values found by Cheney et al.^32^ (melting point 166 °C). Rietveld refinement was used to confirm the phase purity, as presented in Figure 6.

**Figure 5:**
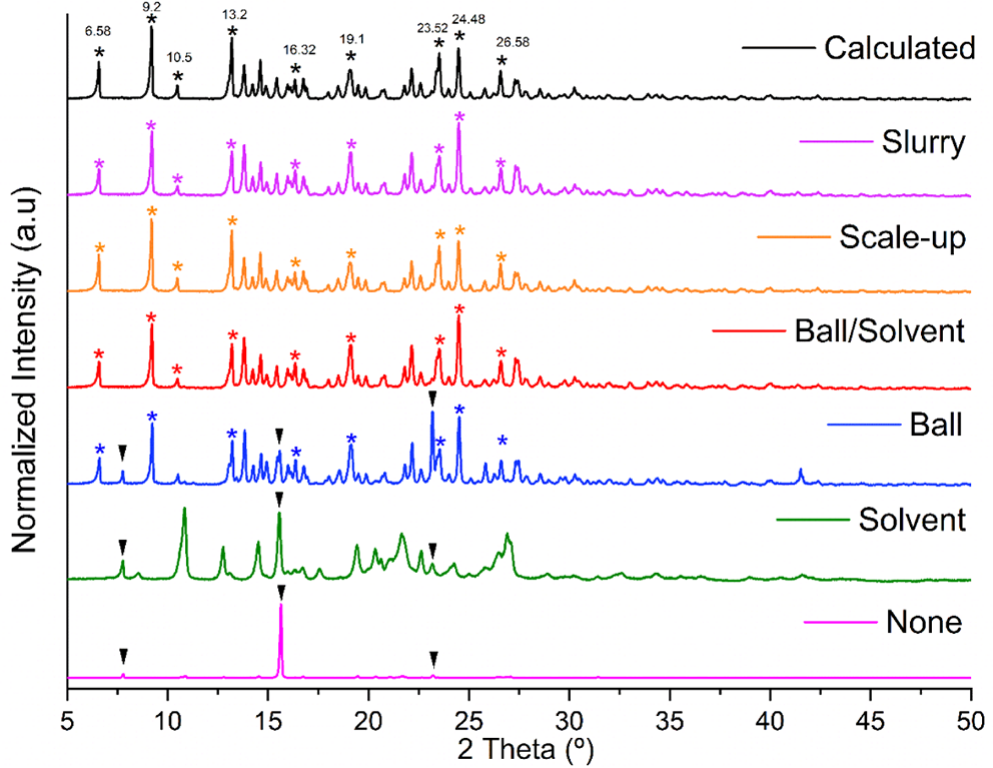
PRXD diffractograms for the validation of the vortex method by reproducing the MEL·AAS cocrystal supramolecular synthesis (black). The experiments were performed using no balls/solvent (pink), no balls/solvent (green), balls/no solvent (blue), and balls/solvent (red). Scale-up (orange) and slurry (purple) experiments were also included.

**Figure 6:**
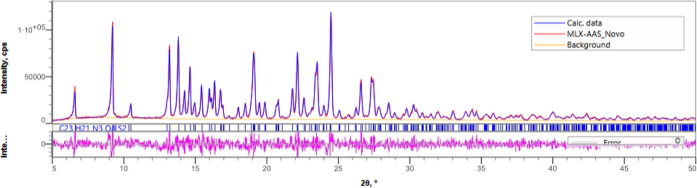
Rietveld refinement plots of the MEL·AAS cocrystal. The blue trace in the plots represents the experimental pattern, the green trace for calculated profile, the orange trace for background, the magenta trace represents the residual between the calculated and observed patterns, and the tick marks are indicative of *hkl* values of the crystal structures. The three samples are found to be phase pure.

As mentioned, slurry (Figure 5 – purple diffractogram) and scale-up (Figure 5 – orange diffractogram) analyses were also performed for the MEL·AAS cocrystal. For the slurry, Cheney et al. kept the system overnight, sealed, and after filtering and washing the resulting solid, the cocrystal was isolated in 96% yield.^32^ By applying the same experimental conditions, but using the vortex-slurry method (balls, 3000 rpm, and opened), it took a total of 52 min to evaporate the THF, yielding 100% of the cocrystal with no need of filtering and/or washing the resulting solid.

For the scale-up experiment, our group used half the amount of solvent employed by Cheney et al.^32^ for the solvent-drop grinding experiment (40 μL of chloroform), which took 30 min to produce 0.279 mg of the cocrystal with approximately 100% conversion in a SPEX Mixer/Mill 8000 M—being worth mentioning that it previously took us only 5 min to reproduce these conditions with the vortex-slurry method (red diffractogram in Figure 5). In this way, for the scale-up experiment, a total of 5 g was prepared, using 3.3 g of MEL, 1.7 g of AAS, and 3.76 mL of chloroform, all added in a Falcon tube with 5 ball bearings, shaken at 3000 rpm for 40 min, also yielding 100% conversion.^32^ Although a similar time was found in comparison with the solvent-drop grinding experiment, in the vortex-slurry experiment half of the solvent was employed as mentioned above, turning this method greener for solvent waste.

#### Ethionamide/Maleic Acid (ETH·MAL) Salt

In 2016, de Melo et al.^21^ reported three salts involving the antituberculosis drug ethionamide: a saccharinate, a maleate, and an oxalate. Only the maleate, however, was successfully reproduced via mechanochemistry using an MM400 Retsch oscillatory ball mill by adding stoichiometric amounts of ETH and maleic acid and milling for 30 min at 25 Hz with no solvent. In all four validation experiments, equimolar amounts of ETH (0.1 mmol) and maleic acid (0.1 mmol) were used in an Eppendorf tube. For the experiments with solvent, a total of 15 μL of ethanol was used. By analyzing Figure 7, it is possible to observe that the ETH·MAL salt was obtained in three of the four validation methods. Table 1 exhibits the time for the reaction for this salt. Particularly, for the validation method using balls and solvent, only 20 s were necessary to salt conversion. This was the only experiment that allowed us to perform a more precise reaction time measurement because ETH is a yellow powder and when it reacts with maleic acid to form the salt, the final powder turns dark orange. The powder diffraction patterns show a very small peaks addressing the presence of pure ETH mixed with the salt. One explanation for this occurrence could be related to the fact that a small fraction of ETH, which is a very thin powder, got stuck into the top of the vessel, thus becoming unavailable to react with maleic acid. In this way, this not reacted amount of ETH was dragged with the sample to the PXRD analyzer. To answer this question, a slurry experiment was performed by pouring solvent to the system to wash the walls before shaking the system. As can be seen in Figure 7, the X-ray diffraction patterns of the solid (orange) indicate 100% conversion into the maleate salt. To confirm the full conversion, additional thermal analysis of the synthesized salt was performed (melting point 139.64 ± 2 °C, Figure S3), agreeing with the values found by de Melo et al.^21^ (melting point 138.32 °C), and Rietveld refinement phase purity (Figure 8).

**Figure 7:**
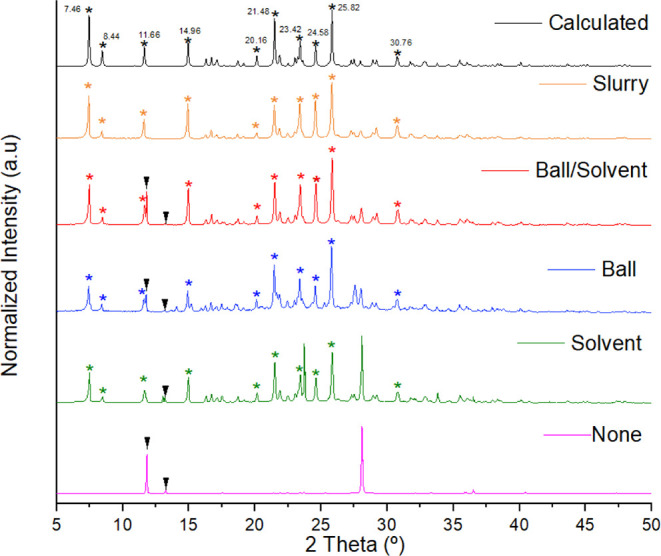
PRXD patterns of the solid obtained by the different methods of synthesis of the ETH·MAL cocrystal (black). The experiments were performed using none balls/solvent (pink), no balls/solvent (green), balls/no solvent (blue), and balls/solvent (red).

**Figure 8:**
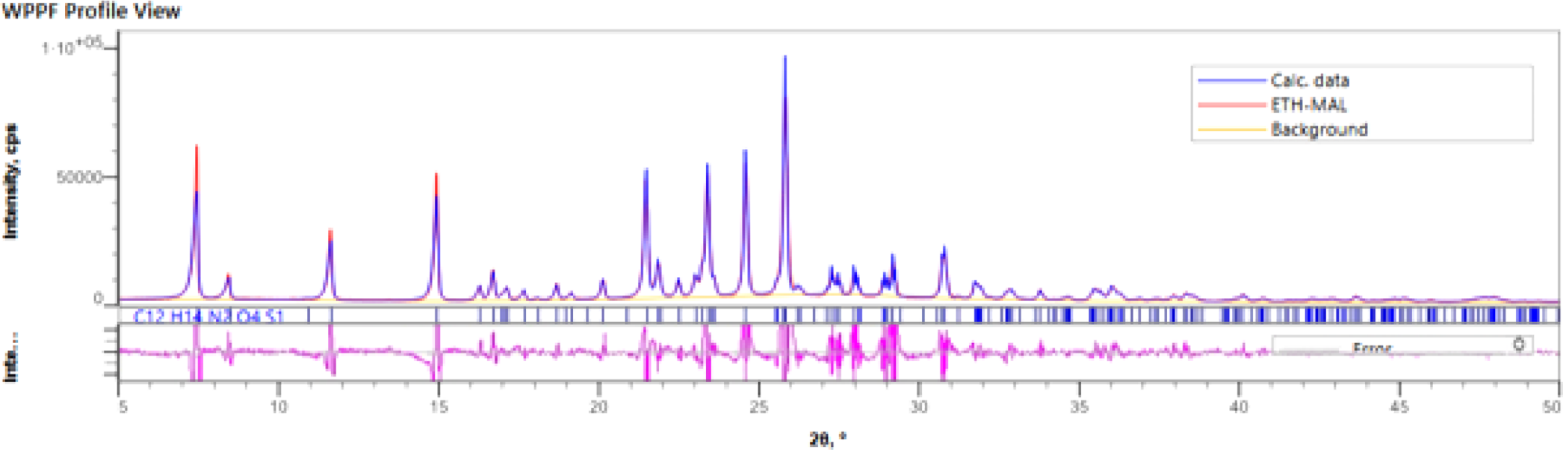
Rietveld refinement plots of the ETH·MAL salt. The blue trace in the plots represents the experimental pattern, the green trace for calculated profile, the orange trace for background, the magenta trace represents the residual between the calculated and observed patterns, and the tick marks are indicative of *hkl* values of the crystal structures. All three samples are found to be phase pure.

**Table 1:** Reaction Time to Reach ETH·MAL Salt Formation^a^ ^a^Time was stopped right after the system turns from yellow to orange.

Reaction time	Validation experiment
5 min	NONE
1 min 30 s	SOLVENT
4 min	BALL
20 s	BALL/SOLVENT
6 min	SLURRY

### Crystal Structure of Hydrated Stavudine Cocrystal with L-Proline

Stavudine [1-(2,3-dideoxy-β-D-glycero-pent-2-enofuranosyl)­thymine] is a synthetic thymidine nucleoside analog with inhibitory activity against HIV/AIDS, besides being classified as BCS I according to the World Health Organization (WHO) and European Medicines Evaluation Agency (EMEA) guidance (E). Stavudine (DT4) does not present an extensive metabolism, being mainly eliminated by renal excretion as an unaltered drug. A review of the literature reveals that some solid forms of DT4 were reported like polymorphs and solvates, with some cocrystals also depicted in other academic work.^41–48^ However, we are unaware of any attempts to generate new cocrystals of DT4 looking for its application in a pharmaceutical solid dosage. So, in this paper we explored the vortex-slurry method to screen a new multicomponent solid form of this drug, a cocrystal of DT4 with L-proline, which showed to be a hydrate, thus named as DT4·Lpro·H_2_O. By considering DT4 extensive metabolism and its high liver damage, the new cocrystal was designed to alleviate the collateral damage by coupling the amino acid L-proline, which is a liver protective compound.^49^ The hydrate formation was not expected but is still a good choice in terms of minimizing the solubility of DT4, thus promoting a more controlled metabolism.

To evaluate the formation of DT4·Lpro·H_2_O, the product generated with the vortex-slurry method was analyzed by PXRD, as shown in Figure 9. The product was also recrystallized in ethanol, where single crystals were obtained for the technique.

**Figure 9:**
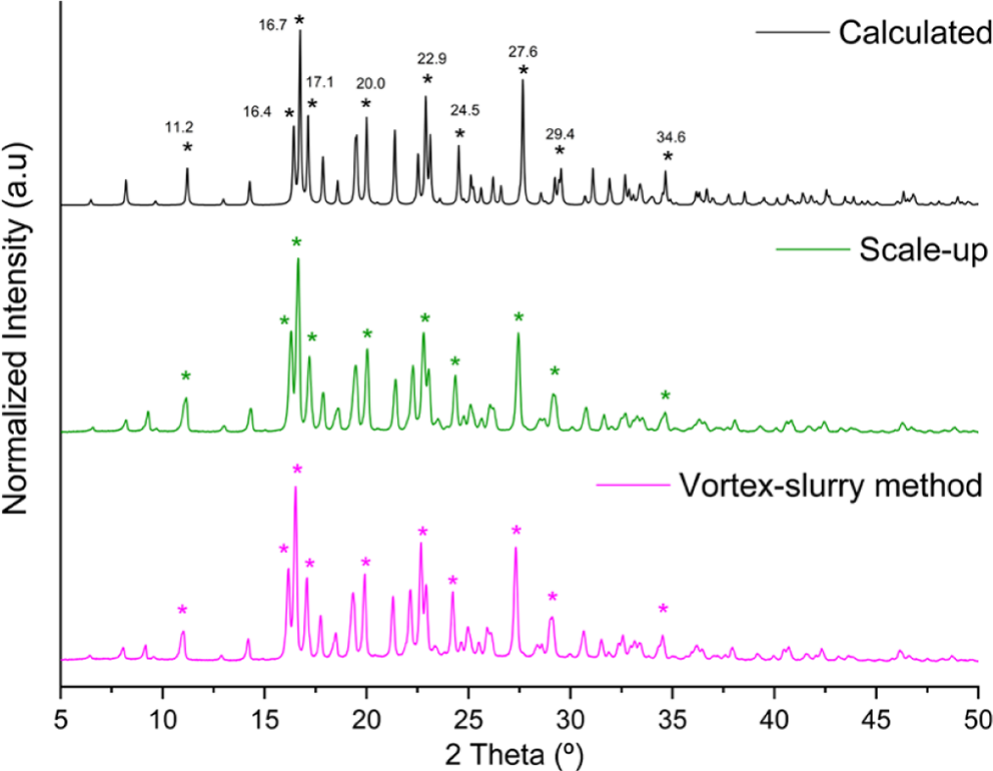
PRXD patterns of the solid obtained by the vortex-slurry method of the DT4·Lpro·H_2_O cocrystal. The experiments were performed using balls/solvent (pink), balls/solvent and scale-up to 3 g (green), and the calculated pattern (black).

The cocrystal crystallizes in the monoclinic space group P2_1_ and has an asymmetric unit composed of one molecule of stavudine, one molecule of L-proline, and one crystallization water molecule, confirming the formation of the hydrate cocrystal with the component molecules in an equimolar ratio. Figure 10 shows the ORTEP-3 ^50^ type representation of the asymmetric unit of DT4·Lpro·H_2_O, with an atom-labeling scheme. Table 2 describes the crystallographic data.

**Figure 10:**
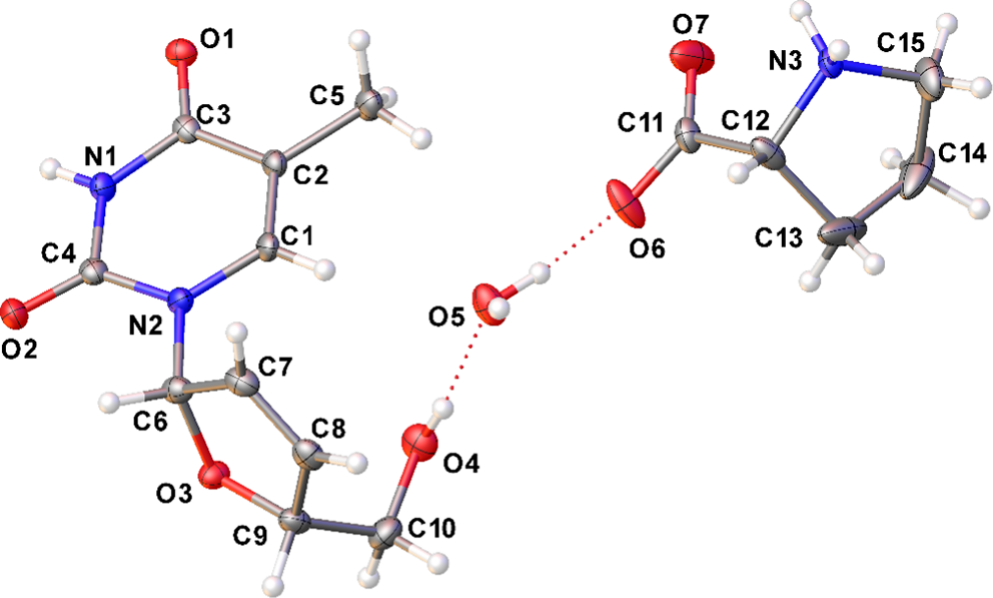
ORTEP-3^50^ type representation of DT4·Lpro·H_2_O, with thermal ellipsoids at 50% of probability.

**Table 2:** Crystal Data and Structure Refinement Parameters for DT4·Lpro·H_2_O

Molecular formula	C_15_H_23_N_3_O_7_
Formula weight	357.36
Temperature (K)	100
Crystal system	Monoclinic
Space group	P2_1_
Unit cell dimensions (Å/°)	*a* = 10.900(2)
	*b* = 5.590(1)
	*c* = 13.790(2)
	β = 98.766(9)
Volume (Å^3^)	830.4(2)
*Z*	2
ρ_calc_ (g/cm^3^)	1.429
2θ range for data collection (°)	2.818 to 47.970
Index ranges	–13, 13; −6,6; −16;16
Reflections collected	10,220
Goodness-on-fit on *F* ^2^	1.043
*R* [*I* > 2σ(*I*)]; *wR*_2_	*R*_1_ = 0.0393, *wR*_2_ = 0.0936
*R* (all data); *wR*_2_	*R*_1_ = 0.0454, *wR*_2_ = 0.0971
Δρ_max_; Δρ_min_ (e Å^–3^)	0.18/–0.22
Flack parameter	–0.3(9)

The electron density map analysis indicates that there is no proton transfer between the DT4 and the L-proline molecules, confirming that the obtained form is a cocrystal. As previously verified, the L-proline molecule is in the neutral zwitterionic form, with the prototropism occurring from the carboxylic acid group to the N3 atom of the cyclic amine.^51^ The furanose and pyrimidine rings are both planar, with r.m.s. deviations of 0.013 and 0.008 Å, respectively, showing a dihedral angle of 75.41° in relation to each other.

The crystal structure of DT4·Lpro·H_2_O is stabilized by the O–H···O and N–H···O hydrogen bonds (Table 2), which organize the molecules in a two-dimensional supramolecular arrangement, as shown in Figure 11a. The N3–H3a···O6 and the N3–H3b···O7 interactions form chains of Lproline molecules (Figure 11b) with $R_{3}^{3}\left(11\right)$ motifs, while the N1–H1···O1 interactions organize the stavudine molecules in chains (Figure 11c). Both chains are connected to each other by the interactions of the O5–H5d···O6 and the O5–H5e···O3 involving the water molecules, with these chains growing along the 2_1_ screw axis in the [010] direction.

**Figure 11:**
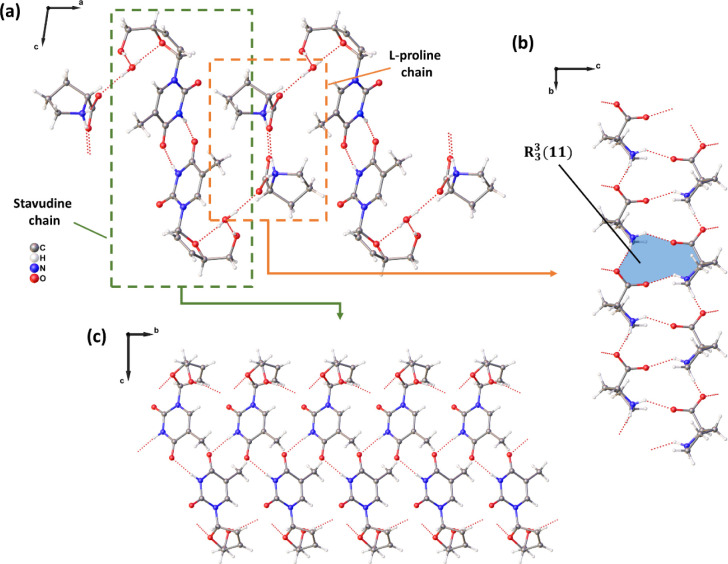
Two-dimensional supramolecular arrangement obtained for the DT4·Lpro·H_2_O cocrystal viewed along the [010] direction **(a)**, and the one-dimensional chains formed for L-proline **(b)** and stavudine **(c)** molecules through intermolecular hydrogen bonds.

The comparison of the theoretical diffractogram obtained for the hydrate cocrystal with the diffractograms generated from the powder formed with the slurry-vortex method reveals the total conversion of the stavudine and L-proline into the hydrate cocrystal, showing the successful application of the proposed methodology to obtain the new cocrystal, even using different quantities of the starting materials.

The thermal behavior of DT4·Lpro·H_2_O was studied using a combination of DSC/TGA techniques. The DSC curve shows no endothermic events associated to the presence of Stavudine or L-Proline, highlighting the total conversion of the experiments in the new cocrystal. The DSC curve, presented in Figure 9, exhibits an endothermic peak at 133.3 °C, being associated to the melting point of the cocrystal, considering that Stavudine (171 °C)^47^ and L-proline (230 °C)^51^ melting points are higher than the one observed. The TGA curve, in turn, shows a mass loss of about 5% around 80.23 °C, which is associated with the water loss, and other events at 159.3, 213.3, and 270.4 °C, associated with the degradation process of the cocrystal. Hot-stage microscopy was performed to corroborate the observed changes in the DSC-TGA experiments. By the images in Figure 12, it is possible to observe change of crystallinity in the cocrystal given the water loss, and decomposition from 159 °C until 300 °C.

**Figure 12:**
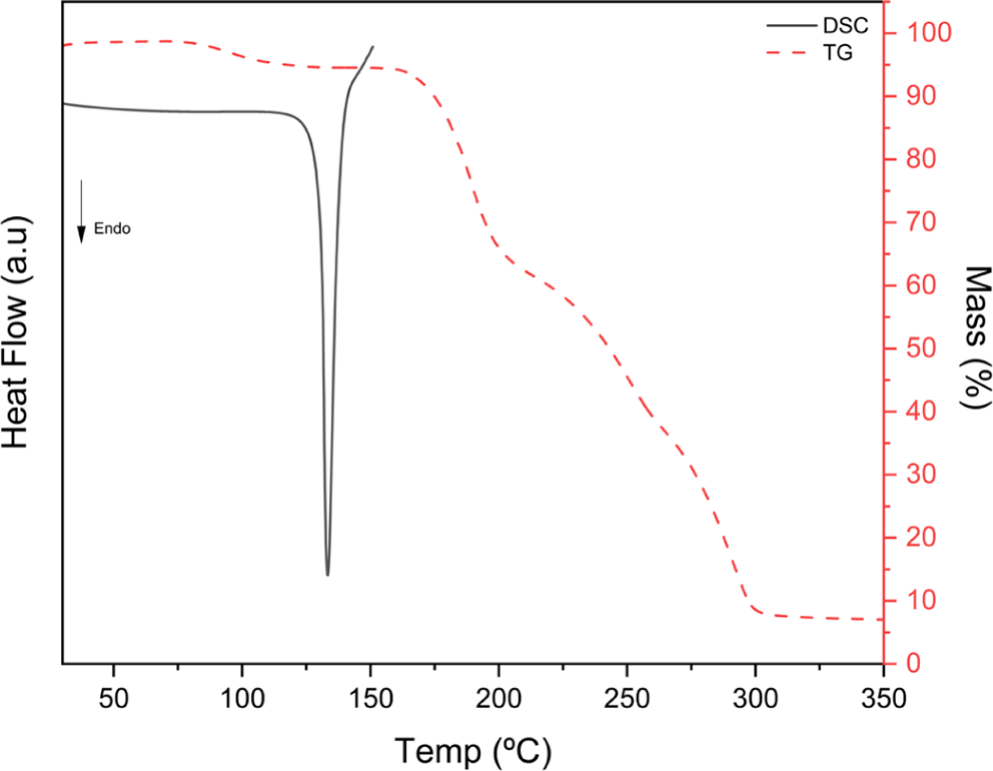
DSC and TGA curves obtained for the DT4·Lpro·H_2_O cocrystal.

### Theoretical Framework for the Vortex-Slurry Method

Spikes^52^ describes the effectiveness of mechanochemistry in the context of the formation of bonds through the lenses of bond stretching and subsequent lowering of activation energy. This happens because the bonds are easier to break in this context. In an analogous way, the effectiveness in supramolecular chemistry can be described as activation energy optimization. We get a lower energy form by deforming the intermolecular bonds of the crystal instead of the covalent bonds. From that point of view, it becomes clear that this should be even easier. Crystal deformations by mechanical collisions probably lower the activation energy, facilitating an almost direct solid state–solid state phase transition. As the crystals are small enough, the repeated collisions damage the crystal, which lowers the energy transition barrier and promotes the self-organization behavior of crystals in a way to minimize the internal energy of the new crystal. The Bell model^53^ subtracts a work term from the activation energy of a bond rupture process. In this work, the concept of bond rupture will be generalized to a growth unit (in classical terms a synthon) leaving the crystal lattice and recombining into a new growth unit or aggregating by itself in the new crystal (crystals can have more than one growth unit).

$$\Delta E_{\mathrm{Bell},F>0}^{‡}=\Delta E_{F=0}^{‡}-F\Delta \xi$$

with,

$$F\Delta \xi \equiv W_{ext}$$

In the same way we define the externally provided work for removing a synthon and reconfiguring it in the new solid form. The Arrhenius activation rate can provide a good framework, already used in the literature^54^ for modeling mechanochemical cocrystallization:

$$k\left(T\right)=Ae^{-E^{‡}/\left(RT\right)}$$

Taking the rate *k* constants, which can be measured using the reaction rate:

r=k(T)⌊A⌋·⌊B⌋

The reaction rate in this case is experimental and measured by taking the derivative of the molar concentration with relation to time, obtained from Raman spectroscopy or a similar technique. Measuring the *k*(*T*) for various temperatures it is possible to determine the activation energy of the entire system:

$$lnk=-\frac{E^{‡}}{RT}+lnA$$

It is still a challenge to determine the exact activation energy in the experimental setup. But, the extremely high conversion rate reported herein shows that the experimental setup is more than enough to overcome such a barrier. Experimental data in a future work may provide enough detail to better correlate the activation energy *W*_ext_ with the frequency of the vortex.

The vortex slurry speed advantage is hypothesized to come from the fact that the chaotic nature of linear and rotational oscillations within the vortex apparatus avoids points of local minima in the flow. This may also be further explored in future work. The diagram below (Figure 13) shows the proposed changes caused by the changes in activation energies.

**Figure 13:**
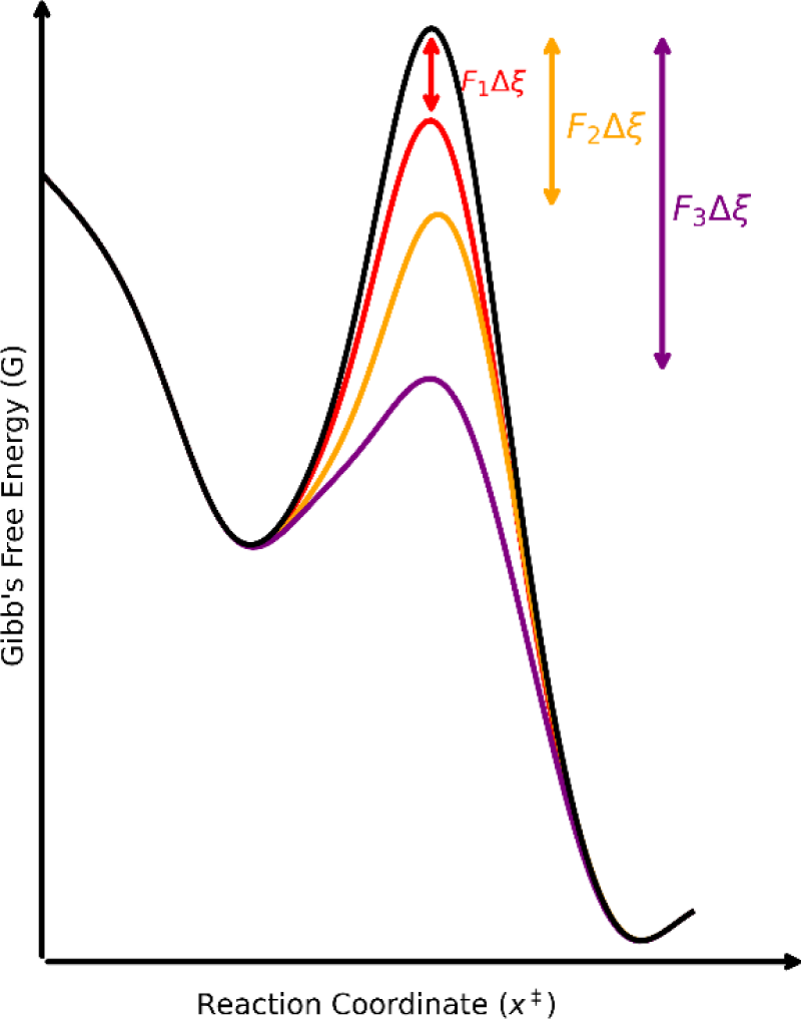
Diagram of the proposed changes caused different activation energies.

#### The Difference in Lattice Energies

The lattice energy^55^ concept was first applied to ionic crystals like rock salt and it can be defined as:

$$dU_{\mathrm{lattice}}=dH_{\mathrm{lattice}}-PdV_{m}$$

where *dU*_lattice_ is the Lattice Energy, *dH*_lattice_ is the lattice Enthalpy, and *dV*_m_ is the change on molar volume due to the formation of the solid state. The system is in fact thermodynamically preferable, as our energy summation simulations demonstrate. Basically, the lattice energy is calculated by direct space summation using OCC.^56^ As defined in Taylor et al.,^56^ the relative stability of a cocrystal is given by:

$$\Delta E_{co‐cryst}=E_{lat}\left(A_{m}B_{n}\right)-\left[mE_{lat}\left(A\right)+nE_{lat}\left(B\right)\right]$$

In Table 3 it is possible to see the results of the simulations using CE-1P.

**Table 3:** Simulations Using CE-1P

	L-Proline	Stavudine	STV+PRL	ΔE_*co‑crystal*_ *(KJ · mol* ^*‑1*^ *)*
*n* (%)	0.339	0.661	1	
*E*_lat_ (kJ · mol^*–*1^)	–236.800	–299.669	–317.317	–38.96

This shows that the cocrystal is very favorable. But being favorable is not necessarily good enough to create a cocrystal. But as the Δ*E*_co‑crystal_ is very high, the mean in the literature^57^ is −19.1 kJ·mol^–1^ maybe it is worth exploring possible correlations between high activation energies and high stability.

## Conclusions

In this work, we report a new implementation for pharmaceutical salts and cocrystals production (via supramolecular synthesis) using a vortex mixer. Two main differences adopted were the bottom tapered vials that alter the ball milling movements in a fashion of increasing the activation energy of the system and the opened vials to allow faster solvent evaporation. Experiments for the validation of the method were performed by resynthesizing three pharmaceutical materials from the literature and by obtaining one new cocrystallization result. In all the experiments, it was possible to show that the newly proposed methodology is reliable, favors scale-up, avoids temperature decomposition, improves production control, and homogenizes the final yield. In addition, the method depicted here was able to generate the new materials in a relatively small reaction time, as well as able to synthesize significant amounts of them (from milligrams to grams), evidencing the possibility of scalability potential (grams to kilograms) once proper modifications are performed. Although it showed to be a very positive implementation, it exhibits limitations, such as the need to construct a better safety mode for allowing solvent to evaporate, adapt manners of allowing the system to be operated without the need of holding the tube, and adapt the system for operating with bigger vessels.

## Supplementary Material





## Notes

(CIF)
DSC curves obtained for HCT·NIC (Figure S1), MEL·AAS (Figure S2), and ETH·MAL (Figure S3) (PDF)
