# Supplementary material for: The Vortex-Slurry Implementation: A Cheap, Easy, and Ultrafast Mechanochemical Tool to Synthesize/Screen Pharmaceutical Salts and Cocrystals
Source: ACS Omega. 2025 Sep 10;10(43):51186–96. doi: 10.1021/acsomega.5c02408 (PMC12593158; doi:10.1021/acsomega.5c02408)
Supplement: Supplementary file 2 [file ao5c02408_si_002.pdf]

THE VORTEX-SLURRY IMPLEMENTATION: A CHEAP, EASY, AND ULTRAFAST MECHANOCHEMICAL TOOL TO SYNTHESIZE/SCREEN PHARMACEUTICAL SALTS AND COCRYSTALS

Paulo N. de Souza[a], *Lucas Vidal C Militão*[a], *Pollyana P. Firmino*[a,b], *Pedro H. de O. Santiago*[a], *João H. de Araujo-Neto*[a,c], *Javier Ellena*[a], *Cecilia C. P. da Silva*[a]*

[a] Universidade de São Paulo, Instituto de Física de São Carlos, Department of Physics and Interdisciplinar Science, Avenida Trabalhador Sãocarlense, 400, Centro, LaMuCrEs, São Carlos, SP, BR 13560-970. [b] Università Di Bologna, Dipartimento di Chimica "Giacomo Ciamician", Via Selmi, 2, Bologna, Emilia-Romagna, IT 40126 ; [c] Universidade de São Paulo, Instituto de Química, Departamento de Quimica Fundamental, Av. Prof. Dr. Lineu Prestes, 748, São Paulo, BR 05508-900

*Corresponding author: cecycarol@yahoo.com.br

Contents of the Supporting Information File

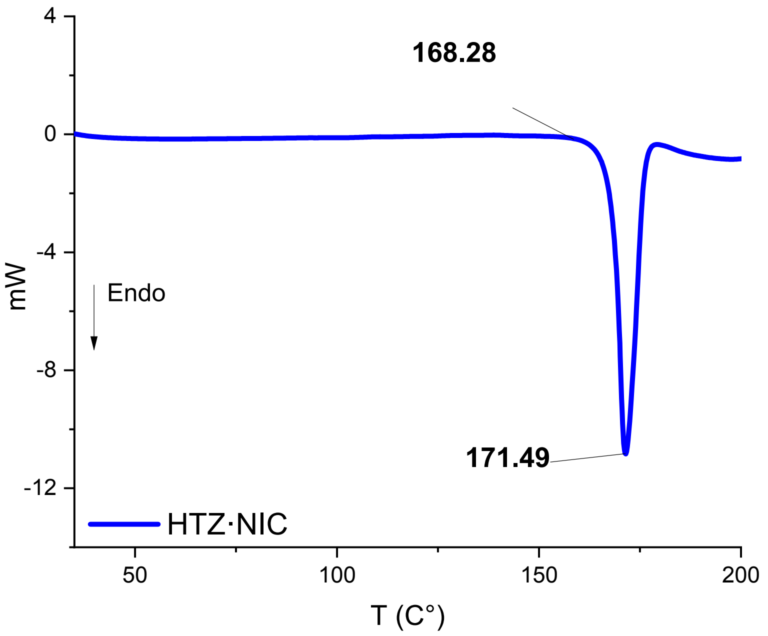

Figure S1. DSC curve obtained for HTZ·NIC cocrystal.

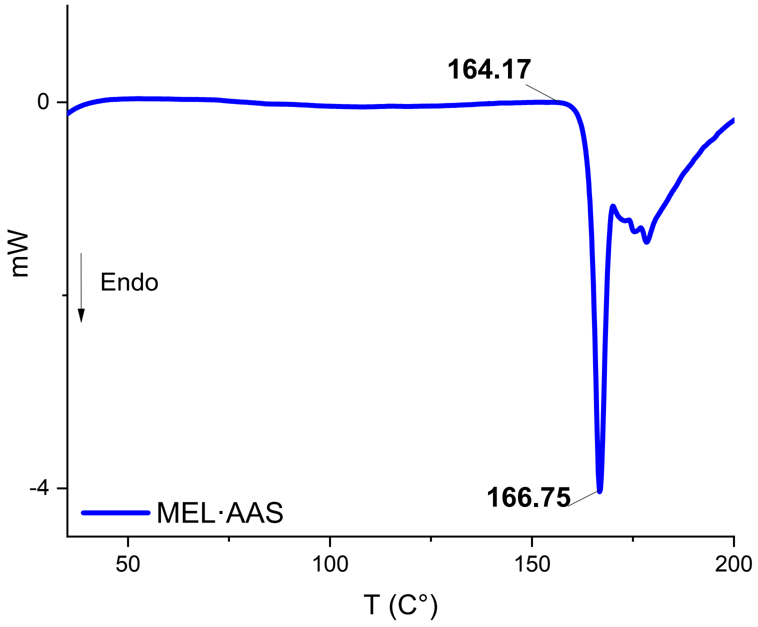

Figure S2. DSC curve obtained for MEL·AAS cocrystal.

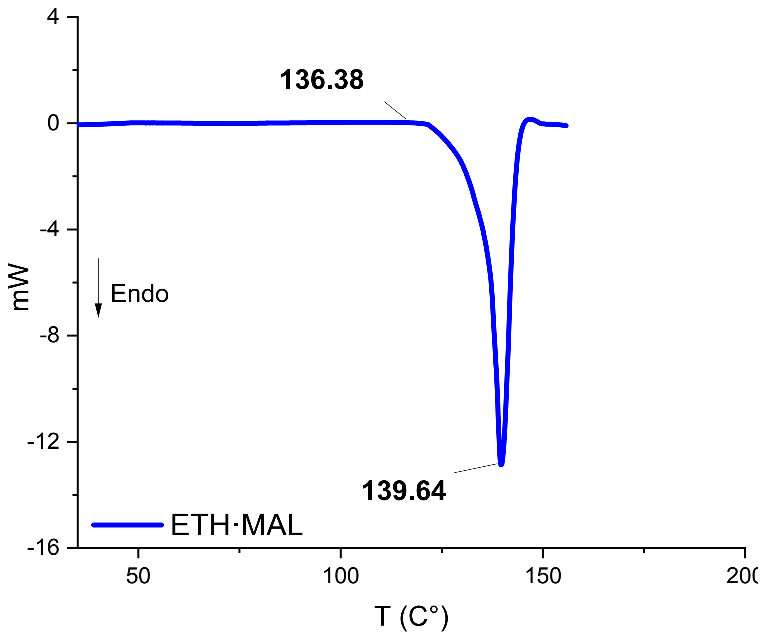

Figure S3. DSC curve obtained for ETH·MAL salt.
